# Comparative Transcriptome Profiling of Cassava Tuberous Roots in Response to Postharvest Physiological Deterioration

**DOI:** 10.3390/ijms24010246

**Published:** 2022-12-23

**Authors:** Ruimei Li, Shuai Yuan, Yangjiao Zhou, Shijia Wang, Qin Zhou, Zhongping Ding, Yajie Wang, Yuan Yao, Jiao Liu, Jianchun Guo

**Affiliations:** 1 Key Laboratory of Biology and Genetic Resources of Tropical Crops, Institute of Tropical Bioscience and Biotechnology, Chinese Academy of Tropical Agricultural Sciences, Haikou 571101, China; 2 Key Laboratory for Biology and Genetic Resources of Tropical Crops of Hainan Province, Hainan Institute for Tropical Agricultural Resources, Haikou 571101, China; 3 College of Tropical Crops, Hainan University, Haikou 570228, China

**Keywords:** cassava, postharvest physiological deterioration, storage, shelf life, transcriptome analysis

## Abstract

Cassava is one of the most versatile tuberous-root crops on Earth. However, the postharvest storage properties of cassava tuberous root mean that it is perishable through a process known as postharvest physiological deterioration (PPD), which seriously affects its starch quality. Therefore, a comprehensive understanding of the transcriptional regulatory activity of cassava against the PPD response is necessary in order to extract key molecular mechanisms related to PPD tolerance. In this study, we found that RYG1 tuberous roots showed delayed PPD compared to those of SC8. In addition, RYG1 roots maintained a more stable cell wall structure after storage than those of SC8. The transcriptome changes in tuberous roots were analyzed for both RYG1 and SC8 after 21 days of storage (SR and SS) compared to fresh (FR and FS) by the RNA-Seq method. The total number of differentially expressed genes (DEGs) in the various comparisons of these four samples ranged from 68 to 3847. Of these, a total of 2008 co-DEGs in SR vs. SS were shared by either SR vs. FR or SS vs. FS. GO and KEGG enrichment analysis revealed that upregulated co-DEGs in SR vs. SS were mainly enriched in photosynthesis, protein processing, hormone and cutin, suberine and wax biosynthesis. By contrast, the downregulated co-DEGs were mainly related to cell wall organization, starch and sucrose metabolism, galactose metabolism, phenylpropanoid biosynthesis, diterpenoid biosynthesis, cysteine and methionine metabolism and flavonoid biosynthesis. The protein–protein interaction (PPI) networks of the co-DEGs showed a complex interaction of genes in different pathways, and 16 hub genes were characterized to have a degree in excess of 15, among which eight genes were associated with photosynthesis. These results provide new information for the study of cassava resistance to PPD and lay a foundation for the further molecular breeding of storage-tolerant cassava varieties.

## 1. Introduction

Cassava (*Manihot esculenta* Crantz) is a starch-rich tuberous-root crop, grown in tropical and subtropical regions, that plays an important role in global food security [1,2,3,4]. The cassava can be used as a food and vegetable for human consumption—similarly to potatoes. It also has value in other applications, including use as livestock and poultry feed [5,6], biofuel production [7,8] and medicine [9]. However, its processing and utilization are seriously affected by its deterioration after harvest, which is known as postharvest physiological deterioration (PPD) [10]. Decay and metamorphism reduce the transparency of starch, seriously affecting the quality and the processing of starch and fuel ethanol, which causes both farmers and industrial enterprises to suffer huge economic losses. Globally, yield losses due to PPD are estimated at up to 29% [10,11]. This has led to a great loss of enthusiasm among the participants in the cassava industry chain. It is estimated that if the storage life of cassava was extended to 45 days, there would be an additional economic benefit of at least USD 35 million per year in Thailand [12,13]. In Africa, the main cassava-producing region, the economics of extending cassava’s shelf-life are expected to be even higher [13]. Therefore, one of the main objectives of cassava breeding is to delay the postharvest rot and thereby improve the shelf life of cassava.

Recognizing the economic impact of postharvest decay, researchers have carried out numerous studies on the storage shelf life of cassava. Different storage techniques have been developed to keep cassava fresh. However, the problem remains unsolved, because the storage techniques used are either too labor-intensive or are limited by increased cassava production [14]. The breeding of new, durable cassava varieties would provide a permanent solution to the perishability problem affecting cassava after harvest. In 2002, Coters et al. identified 10 genes related to the wound response in cassava PPD using the quantitative trait loci (QTL) mapping method [15]. Genome-wide association studies (GWAS) are another potential genetic anatomical method for complex traits. Although the role of GWAS in cassava PPD has not been reported, GWAS studies on cassava root rot have been reported [16]. It can also provide a reference for the molecular mechanism of cassava postharvest metamorphism. Genomic selection (GS) is also a very important technique in plant breeding. At present, it has been applied in cassava breeding [17,18,19,20,21], but its application in cassava PPD has not been reported. With the development of biotechnology and improvements in cassava genetic transformation systems, it is possible to obtain decay-resistant cassava varieties by means of genetic engineering. Obtaining a fuller understanding of the molecular mechanisms of cassava decay and deterioration after harvest is therefore of great significance in the cultivation of cassava varieties with resistance to PPD via molecular means.

Because agricultural products respond to postharvest storage stress in a species-specific manner, transcriptome sequencing has been widely used to study postharvest gene expression in different agricultural products and to understand the molecular mechanisms of PPD and resistance to PPD [22,23,24,25,26,27,28]. Transcriptome analysis has also been carried out in several cassava PPD studies. Huang et al. (2001) used an RNA fingerprinting method, called cDNA-AFLP, to study the gene changes and important metabolic pathways in the early stage (the first 72 h) of PPD in cassava. Seventy transcription-derived fragments were screened and isolated. The analysis showed that important biochemical and physiological processes such as oxygen stress, carbohydrate metabolism, protein metabolism and phenolic compound synthesis were involved in PPD in cassava [29]. Reilly et al. (2007) analyzed the transcriptome of cassava roots after harvest and identified 72 genes that were altered during postharvest regulation. Many of the upregulated and PPD-specific expressed genes are involved in cellular processes such as reactive oxygen species turnover, cell wall repair and programmed cell death [30]. Transcriptome analysis has also been used to analyze the role of melatonin in delaying the PPD of cassava [31,32]. Melatonin treatment activated the expression of several antioxidant enzymes, calcium signaling, the MAPK cascade and transcription factors, as well as starch degradation-related genes in cassava [31,32]. Yan et al. (2021) conducted a comparative transcriptome analysis of cassava postharvest deterioration and found that the expression of a large number of genes encoding antioxidant enzymes and protein kinases was significantly upregulated in the process of PPD, and the expression of genes related to starch degradation was significantly upregulated, while genes related to starch synthesis were significantly downregulated [33]. Previous studies have mainly focused on the stress response of cassava stored for a short time after harvest, such as 6 h, 12 h, 48 h [31,32,33], 72 h [29] or the longest period of 11 days [34], but the changes in the gene expression levels of cassava stored for 3 weeks without decay have not been studied. It is more important to identify the key genes that maintain the PPD tolerance in the more storable cassava.

In a previous study, we found that RYG1 cassava tuberous roots were significantly more resistant to PPD than SC8 cassava [35]. In the current study, to further understand the molecular basis for the extended shelf life of RYG1, the transcriptomes of two cassava varieties at two time points after harvest (fresh and after 21 days in storage) were established and compared. Some important genes and pathways related to maintaining a longer shelf life in cassava have been found. The results of this study will provide new theories and insights for extending the shelf life of cassava by molecular means.

## 2. Results

### 2.1. Comparison of the PPD Tolerance of SC8 and RYG1 Cassava Tuberous Roots during Storage

**Figure 1:**
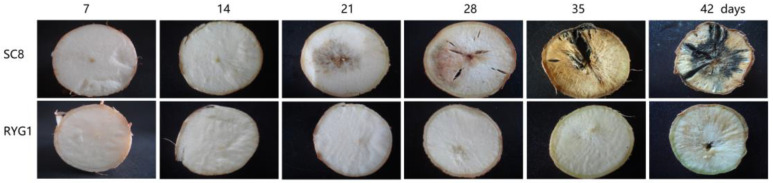
PPD detection results for the SC8 and RYG1 cassavas after the proximal and distal ends were removed.

After comparing the PPD in both SC8 and RYG1 cassava, we found that the RYG1 cassavas were more resistant to PPD than SC8 cassavas (Figure 1). As is apparent in Figure 1, SC8 cassava roots were significantly deteriorated after 21 days of storage and totally deteriorated after 28 days, with fungal growth being clearly observable after 35 days, and, by 42 days, the fungus was spread more widely. By contrast, RYG1 roots remained fresh, without any sign of deterioration after 21 days of storage. Indeed, by 35 days, the peel and flesh of cassava were still be observed to be light green. However, at 42 days, RYG1 cassava roots appeared to change to a light coffee color, which is symptomatic of deterioration (Figure 1). These results indicated that the RYG1 cassava was more tolerant to PPD than SC8.

### 2.2. Microscopic Structure of SC8 and RYG1 Cassava

**Figure 2:**
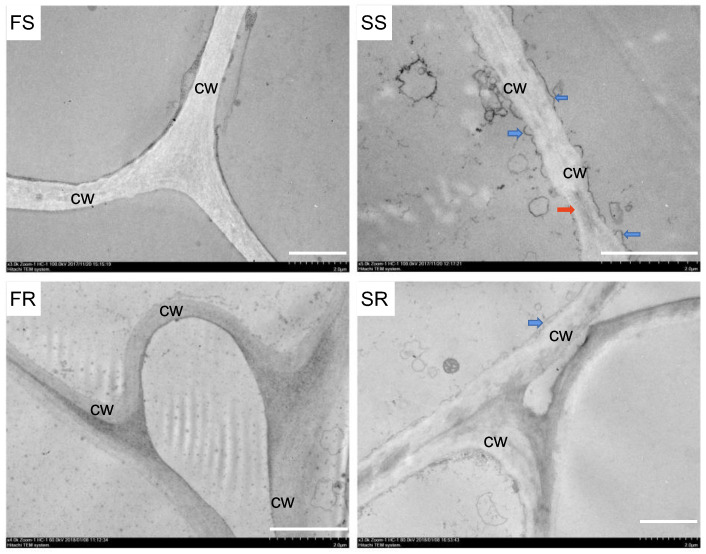
Transmission electron microscopy images of SC8 and RYG1 cassava. CW, cell wall. Blue arrow, plasma membrane invagination. Red arrow, cell wall collapse. The white lines indicate 2 μm.

To understand how the structure of cassava changed after storage, the ultrastructural features of RYG1 and SC8 cassava were observed, both fresh and after 21 days of storage, by transmission electron microscopy. The results revealed that the cell wall and membrane were close together, without separation, both in fresh RYG1 (hereafter, FR) and fresh SC8 (hereafter, FS). The cell wall was smooth and uniform in morphology. By contrast, after 21 days of storage of RYG1 (hereafter termed SR) and 21 days of storage of SC8 (hereafter termed SS), the different cassava cultivars showed great differences in their cell structures. The cell structures of SS were severely damaged, with visible morphological changes, including plasma membrane invagination and cell wall collapse. Meanwhile, in SR, the cell morphological changes were considerably weaker than those exhibited by SS (Figure 2).

### 2.3. Assembly of Transcriptomes and Quality Assessments

**Figure 3:**
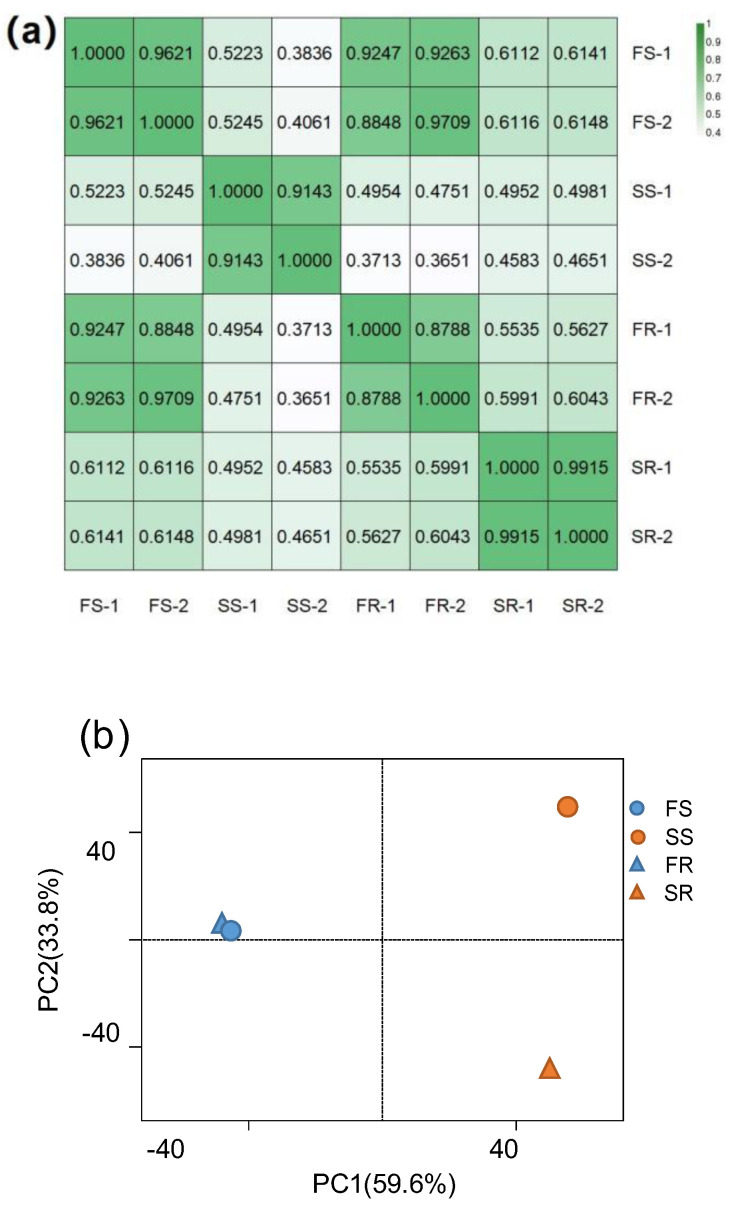
Quality assessment of transcriptomes. (**a**) Spearman’s correlation analysis between biological replicates for RNA-seq. Color levels correspond to the degree of correlation in each replicate. (**b**) Principal component analysis (PCA) of the whole transcriptome in cassava samples.

To determine how the tuberous roots of RYG1 maintain a longer shelf life than SC8, we performed mRNA sequencing to analyze the transcriptomes of tuberous roots from FS, FR, SS and SR. In our data, each sample generated an average of ~2.96 GB of clean data (Table S1). Clean reads with quality scores ≥20% reached more than 95%, and clean reads with quality scores ≥30% accounted for more than 90% (Table S1). After the reads were filtered, 18.48–24.27 million clean reads were obtained, among which more than 73.92% were mapped to the cassava reference genome. We found that 73.57% of the mapped reads were aligned to unique locations. Finally, 31,592 genes were obtained (Table S1). Next, correlation analysis between biological replicates of RNA-seq data showed high Pearson’s correlation values (R^2^ ≥ 0.88) between repeats (Figure 3a), indicating that the sequencing results exhibited high reproducibility.

The total transcriptional responses of FS, FR, SS and SR were next compared. The principal component analysis (PCA) plot showed 59.6% variance in PC1 and 33.8% variance in PC2 (Figure 3b). The FS and FR groups were difficult to separate by either PC1 or PC2, indicating that their global gene expression was highly similar. By contrast, PC1 clearly separated the samples of SS and SR from the FS and FR (Figure 3b), suggesting that the overall gene expression profiles of cassava were largely affected by storage stress when compared with the fresh condition. Notably, PC2 clearly separated the samples of SS and SR from both FS and FR, even separating SS from SR, indicating that highly diverse patterns of gene expression existed between SS and SR, between SR and FR and between SS and FS (Figure 3b).

### 2.4. Identification of Differentially Expressed Genes

**Figure 4:**
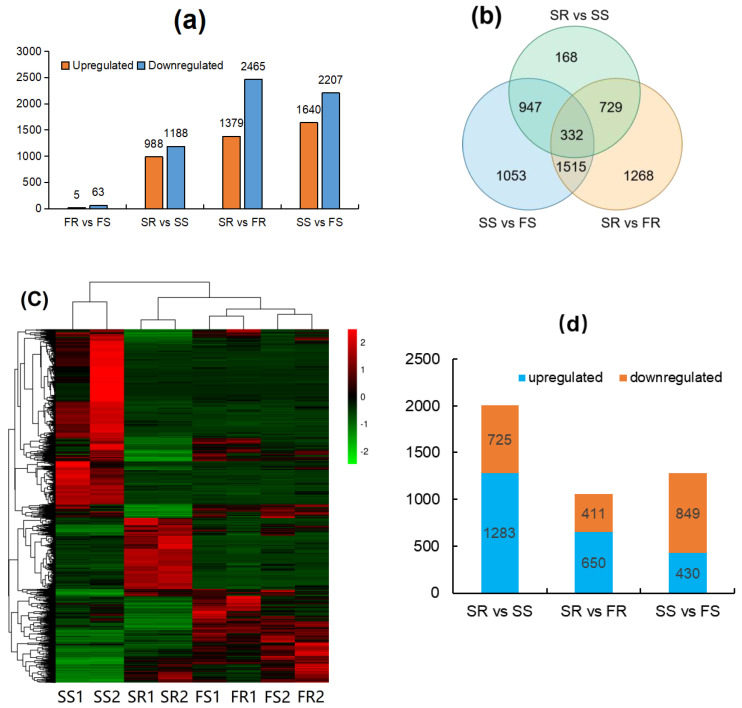
Differentially expressed gene analysis of FR, SR, FS and SS. (**a**) DEGs in statistics for FR vs. FS, SR vs. SS, SS vs. FS and SR vs. FR. The ordinate value represents the number of DEG. (**b**) Venn diagram depicting co-expressed and specific expressed DEGs in the pairwise comparisons: SR vs. SS, SS vs. FS and SR vs. FR. (**c**) Heatmap of the selected 2008 (322, 947 and 729) common DEGs. The color scale represents Z-score. (**d**) The upregulated and downregulated genes in the 2008 common DEGs. The ordinate value represents the number of co-DEG.

**Figure 5:**
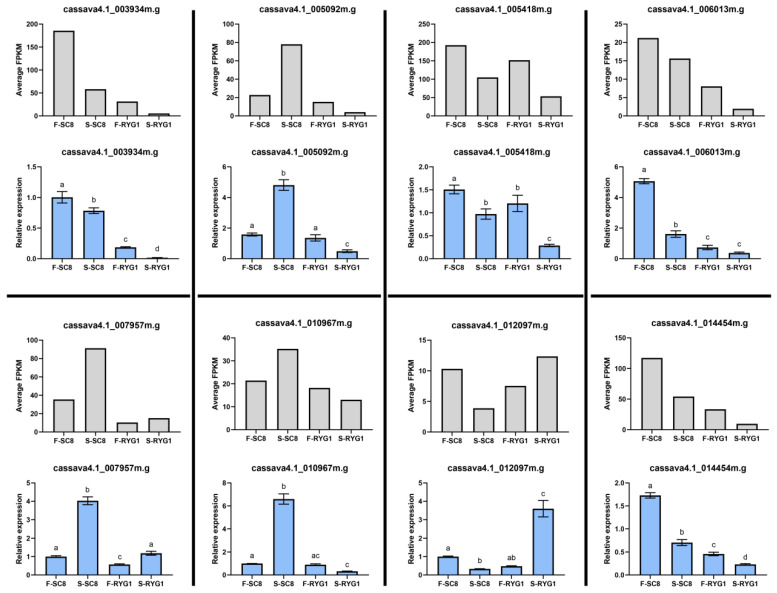
qRT-PCR validation of the RNA sequencing results for selected DEGs. The grey bars represent the FPKM values of genes from RNA-seq. The blue bars represent the values determined by qRT-PCR. The error bars indicate the standard deviation. The characters on the top of each bar indicate a significant difference.

We subsequently analyzed the differentially expressed genes (DEGs) in the four pairwise comparisons with the criteria of |log2(fold change) | more than 2 and false discovery rate (FDR, adjusted *p*-value) less than 0.05. Among them, there were 68 DEGs (five upregulated, 63 downregulated) between FR and FS, 2716 DEGs (988 upregulated, 1188 downregulated) between SR and SS, 3844 DEGs (1379 upregulated, 2465 downregulated) between SR and FR and 3847 DEGs (1640 upregulated, 2207 downregulated) between SS and FS (Figure 4a). These results demonstrate that the storage of cassava led to a larger number of DEGs than those caused by the cassava germplasm variety difference. In order to ensure the accuracy of the RNA-seq data, eight DEGs were randomly selected for the subsequent qRT-PCR validation experiments. The results showed that the expression trends of selected genes obtained by qRT-PCR were basically similar to the RNA-seq results (Figure 5). As such, this result confirms the reproducibility and reliability of the RNA-seq data in this study, which can be used with confidence in subsequent studies.

### 2.5. Venn Analysis of the DEGs

To explore genes that possibly contributed to differences in RYG1 and SC8’s resistance to PPD, we used Venn diagrams to display the common DEGs (co-DEGs) shared by different comparisons. This analysis revealed that there are 332 genes shared by SR vs. SS, SS vs. FS and SR vs. FR; 947 genes only shared by SR vs. SS and SS vs. FS; 729 genes only shared by SR vs. SS and SR vs. FS; and 1515 genes only shared by SR vs. FR and SS vs. FS. Furthermore, there were 168 genes unique to SR vs. SS, 1053 genes unique to SS vs. FS and 1268 genes unique to SR vs. FR (Figure 4b). We subsequently found that a total of 2008 genes in SR vs. SS were shared either with SS vs. FS or with SR vs. FR (Figure 4b). It is speculated that these genes may be the main factors responsible for the differences in response to PPD between the two varieties. Therefore, they were selected for further studies and their expression patterns in both varieties and under both conditions were visualized in a heatmap (Figure 4c). Among the 2008 genes, there were 725 genes upregulated and 1283 genes downregulated in SR vs. SS, 411 upregulated and 650 downregulated in SR vs. FR and 849 upregulated and 430 downregulated in SS vs. FS (Figure 4d).

### 2.6. GO Functional Enrichment Analysis of Co-DEGs

In an attempt to determine the molecular differences in RYG1 and SC8’s response to PPD, we analyzed the functional annotations of the 2008 co-DEGs. The 725 upregulated and 1283 downregulated co-DEGs in SR vs. SS were subjected to GO classification separately. They grouped into three broad categories: biological processes, cellular components and molecular functions (Table S2). In the upregulated gene sets, genes were enriched in biological process categories related to the response to stimuli, such as light, heat, reactive oxygen, salt stress, hormones, alcohol and inorganic substances. These genes were also enriched in categories related to photosynthesis, such as light reaction and light harvesting, and categories related to protein folding. In the downregulated gene sets, genes were enriched in categories related to metabolism processes, such as the cellulose catabolic process, phenylpropanoid biosynthesis, chitin catabolic process, gibberellin biosynthetic process, hydrogen peroxide catabolic process and glutathione metabolic process; in categories related to cell wall organization or biogenesis; and in categories related to stimuli, such as ethylene, jasmonic acid, salicylic acid, fungus, immunity, wounding and oxidative stress (Figure S1).

### 2.7. KEGG Pathway Analysis of co-DEGs

A KEGG pathway enrichment analysis was carried out for both 725 upregulated co-DEGs and 1283 downregulated co-DEGs (Table 1). In the upregulated gene sets, the energy metabolism-related pathways, including photosynthesis and photosynthesis—antenna proteins; protein folding, sorting and degradation-related pathways; protein processing in the endoplasmic reticulum; lipid metabolism-related pathways, including cutin, suberin and wax biosynthesis and fatty acid elongation; and the signal transduction-related pathway—plant hormone signal transduction, were significantly enriched. In the downregulated gene sets, a total of 19 pathways were significantly enriched, which included cysteine and methionine metabolism, phenylpropanoid biosynthesis, flavonoid biosynthesis, carbon metabolism, glutathione metabolism, the MAPK signaling pathway—plants, starch and sucrose metabolism, galactose metabolism and terpenoid backbone biosynthesis.

### 2.8. Differentially Expressed Genes Related to Photosynthesis

**Figure 6:**
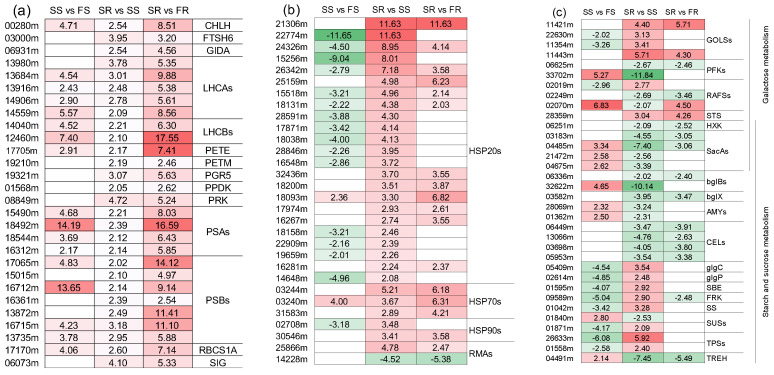

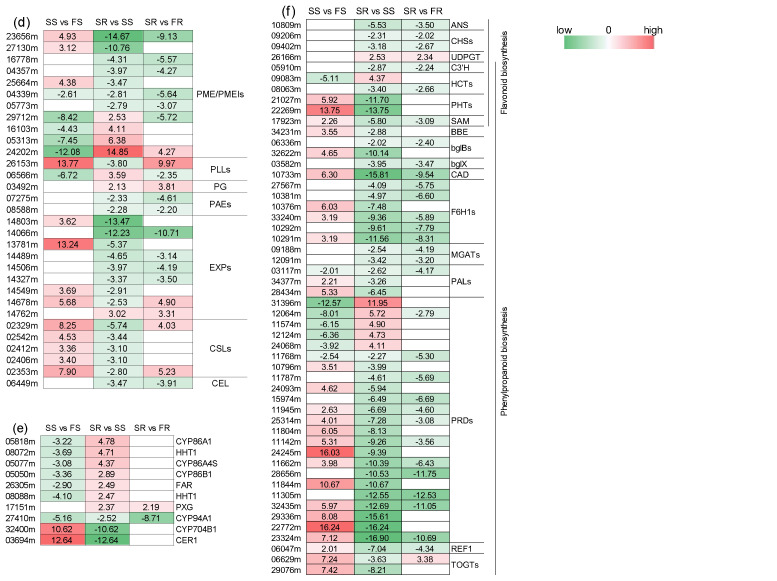
Heatmap of differentially expressed genes related to photosynthesis (**a**), protein processing in endoplasmic reticulum (**b**), galactose metabolism and starch and (**c**), cell wall (**d**), cutin, suberin and wax biosynthesis (**e**) and phenylpropanoid biosynthesis and flavonoid biosynthesis (**f**).

In this study, both the GO and KEGG enrichment analysis of co-DEGs revealed that photosynthesis-associated terms were significantly enriched in the upregulated co-DEGs (Figure S1, Table 1 and Table S2). We therefore searched the 2008 co-DEGs to identify all of the photosynthesis-related genes and their expression patterns. A total of 28 genes that participate in photosynthesis, including *LHCAs*, *LHCBs*, *PETs*, *PSAs*, *PSBs*, *CHLH*, *PRK*, *PPDK*, *GIDA*, *FTSH6*, *PGR5*, *RBCS1A* and *SIG*, were revealed (Figure 6a). It was discovered that all the DEGs had higher expression in stored RYG1 than in stored SC8, even though they were induced in both varieties after storage in comparison to their fresh condition. These results suggest that the stored RYG1 may exhibit higher rates of photosynthesis than the stored SC8. However, whether the photosynthetic process is active remains to be confirmed in future studies.

### 2.9. Differentially Expressed Genes in Protein Processing in the Endoplasmic Reticulum Pathway

Protein processing in the endoplasmic reticulum pathway was another significantly enriched pathway in the upregulated co-DEGs. Here, we further examined all genes in this pathway in co-DEGs, revealing in a total of 30 genes (Figure 6b). There were 23 *HSP20s*, three *HSP70s*, two *HSP90s* and two *RMAs*. All of the *HSP20s*, *HSP70s* and *HSP90s* were upregulated in SR compared SS. Most of them were upregulated in SR compared to FR, while others displayed no significant difference. Meanwhile, in SS vs. FS, most of the *HSP* genes were downregulated. In addition, for the two *RMAs*, one was upregulated and the other was downregulated in both SR vs. SS and SR vs. FR, but there was no difference in SS vs. FS.

### 2.10. Differentially Expressed Genes Related to Starch and Sucrose Metabolism and Galactose Metabolism

A total of 34 co-DEGs were identified from the starch and sucrose metabolism and galactose metabolism pathways, in which five genes (one *HXK*, four *SacAs*) belonged to both pathways (Figure 6c). In addition, four *GolSs*, one *STS*, one *RAF*, one *glgP*, one *glgC*, one *SBE*, one *FRK*, one *SS*, two *TPSs* and two *SUSs* genes were upregulated in SR vs. SS, while the other genes, such as two *PFKs*, one *RAF*, one *HXK*, four *SacAs*, two *bglBs*, one *bglX*, two *AMYs*, four *CELs*, one *SUS* and one *TREH*, were downregulated in SR vs. SS.

### 2.11. Differentially Expressed Genes Related to Cell Wall, Cutin, Suberin and Wax Biosynthesis, Phenylpropanoid Biosynthesis and Flavonoid Biosynthesis

In the present study, we found that genes in the cell wall organization GO term and the cutin, suberin and wax biosynthesis, phenylpropanoid biosynthesis and flavonoid biosynthesis KEGG pathways were significantly enriched in the SR vs. SS comparison. Therefore, we further examined the relative levels of expression of these genes. Notably, 11 *PME/PMEIs* (four upregulated and seven downregulated), two *PLLs* (one upregulated, one downregulated), two *PAEs* (downregulated) and one *PG* (upregulated) were identified from the SR vs. SS comparison, and all of these genes acted on pectin in the cell wall components. In addition, our study revealed that five *CSLs* and one *CEL* gene(s), which are associated with cellulose, were identified, and all of them were downregulated in SR vs. SS. Moreover, a total of nine *EXPs* (one upregulated, eight downregulated) were identified from the SR vs. SS comparison (Figure 6d). For the cutin, suberine and wax biosynthesis pathway, a total of 10 genes (three upregulated, seven downregulated) were identified (Figure 6e). A total of 52 genes were identified from the phenylpropanoid biosynthesis or flavonoid biosynthesis pathways, in which six genes (two *HCTs*, two *PHTs*, one *C3′H* and one *SAM*) belonged to both pathways. Most of the genes (45 out of 52) were downregulated in SR vs. SS, while only seven genes were upregulated in SR vs. SS (Figure 6f).

### 2.12. Differentially Expressed Genes Related to Hormone Signaling

**Figure 7:**
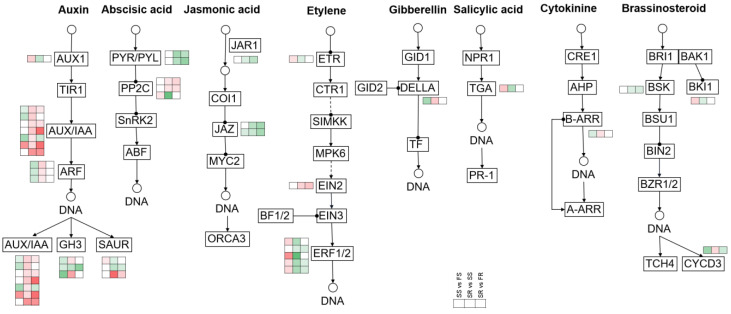
Differentially expressed genes in the plant hormone signal transduction pathway.

To explore how the plant hormones were involved in cassava PPD tolerance, the expression profiles of 38 genes related to plant hormone signal transduction were analyzed (Figure 7 and Table S3). For the auxin signal, 17 genes were differentially expressed in SR vs. SS, thirteen of which were upregulated, including genes encoding seven *AUX/IAAs*, three *ARFs*, two *GH3s* and one *SAUR*. For the ABA signal, two *PP2C* genes were upregulated after storage in SR vs. SS, while another *PP2C* and two *PYLs* were downregulated. For the JA signal, one *JAR1* gene and two *JAZs* were downregulated in SR vs. SS. For the SA signal, only one *TGA* gene existed and was downregulated in SR vs. SS. For the CTK signal, only one *B-ARR* gene existed and was upregulated. For the ETH signal, one *ETR* and five *ERFs* were downregulated, while only one *EIN2* gene was upregulated in SR vs. SS. For the GA signal, only one *DELLA* was upregulated in SR vs. SS. For the BR signal, one *BSK* and one *BKI1* were downregulated, while one *CYCD3* was upregulated in SR vs. SS.

### 2.13. Differentially Expressed Genes Related to Transcriptional Regulation

**Figure 8:**
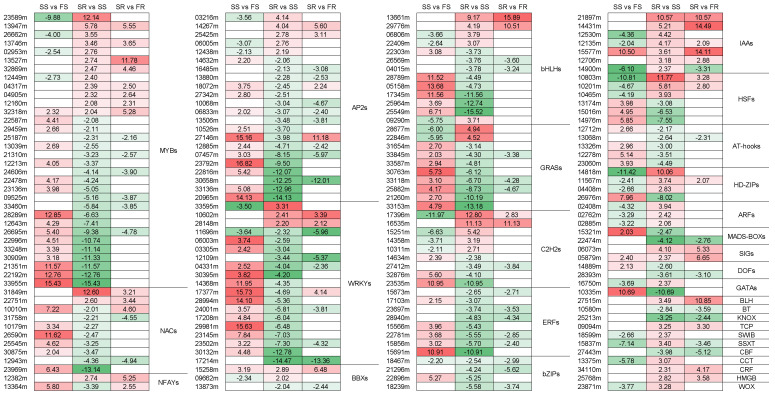
Heatmap of differentially expressed genes related to transcriptional regulation.

Co-DEG analysis indicated that a total of 176 genes encoding putative transcription factors (TFs) were significantly differentially expressed between RYG1 and SC8 after storage, and their expression heatmap is presented in Figure 8. These TFs were categorized into 31 different families, including *MYBs*, *AP2s*, *WRKYs*, *NACs*, *bHLHs*, *GRASs*, *C2H2s*, *HSFs* and *IAAs*. The MYB family of transcription factors was the most abundant (32 genes), followed by the AP2s (22 genes), WRKYs (19 genes), bHLHs (13 genes), NACs (10 genes) and GRASs (10 genes) (Figure 8).

### 2.14. Protein–Protein Interaction Network Construction and Hub Gene Identification

**Figure 9:**
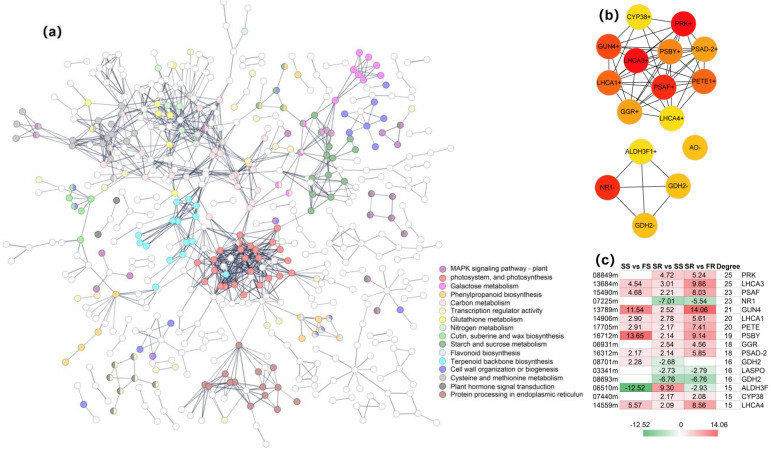
Network analysis of co-DEGs and hub gene identification. (**a**) The networks of co-DEGs. The different colors indicate the diverse pathways that genes participated in. (**b**) Hub genes with edges numbering more than 15 selected from the network of co-DEGs by using cytoHubba plugin in Cytoscape v3.9.0. The color of nodes from red to yellow represents the degree of connectivity of nodes, with red being highly connected and yellow weakly connected. The “+” indicates upregulated genes in SR vs. SS, while the “-” indicates the downregulated genes in SR vs. SS. (**c**) The differential expression of hub genes. The red cells from light to dark indicate upregulation. The green cells from light to dark indicate downregulation. The white cell indicates no significant difference.

As described above, the transcriptome analysis identified a list of co-DEGs, and these genes participated in differential pathways or biological processes. To reveal the interaction relationships among these co-DEGs, a protein–protein interaction (PPI) network was constructed on the basis of gene co-expression. The co-DEGs formed PPI networks with 524 nodes and 1004 edges, in which 324 nodes with 874 edges formed the largest network (Figure 9a). Genes in diverse pathways were intertwined, which illustrated the complexity of cassava’s resistance to PPD. The intertwined genes were associated with pathways such as photosynthesis, protein processing in the endoplasmic reticulum, carbon metabolism, nitrogen metabolism, starch and sucrose metabolism, etc. Next, by running the cytoHubba plugin in Cytoscape v3.9.0, the genes with a degree of connectivity in excess of 15 were identified as hub genes. Thereby, a total of 16 hub genes were obtained, of which the degree of connectivity was 25 for both *PRK* and *LHCA3*, 23 for both *PSAF* and *NR1*, 21 for *GUN4*, 20 for both *LHCA1* and *PETE*, 19 for *PSBY*, 18 for both *GGR* and *PSAD-2*, 16 for *GDH2s* and *LASPO* and 15 for *ALDH3F1*, *CYP38* and *LHCA4* (Figure 9b, c). Among these hub genes, there were 12 hub genes, including *PRK, LHCA3, PSAF, GUN4, LHCA1, PETE, PSBY, GGR, PSAD-2, ALDH3F1, CYP38* and *LHCA4*, that were up-regulated, and four hub genes (*NR1*, 2 *GDH2s* and *LASPO*) were down-regulated in SR vs. SS. In addition, eight of the hub genes, including *PRK, LHCA3, PSAF, LHCA1, PETE, PSBY, PSAD-2* and *LHCA4*, were associated with photosynthesis, which further suggested the crucial role of photosynthesis-related genes in RYG1 cassava’s resistance to PPD (Figure 9c).

## 3. Discussion

Postharvest physiological deterioration (PPD) of cassava is a major obstacle to the development of the cassava industry. It is a complex physiological and biochemical process involving changes in gene expression, protein synthesis and secondary metabolite accumulation [30]. In recent decades, great progress has been made in improving cassava’s resistance to PPD, but it is far from sufficient. Here, we compared the differences in the postharvest response to PPD between RYG1 (PPD-tolerant) and SC8 (PPD-susceptible) cassava cultivars in terms of their phenotype, microstructure and transcriptome. On the whole, the phenotypes and microstructures of fresh RYG1 and SC8 cassavas were significantly different from those at 21 days after harvest under the same storage conditions. The differences in the physiological responses of the two cassava germplasms after storage were partly caused by the differences in gene expression. Therefore, four sets of transcriptomic data were compared to analyze the functional information of differentially expressed genes, using fresh cassava samples (FR and FS) and 21-day-storage cassava samples (SR and SS). From the perspective of the principal component analysis of gene expression, SC8 and RYG1 cassava samples showed little difference in their gene expression profiles when fresh, but had completely different gene expression profiles after storage, presenting a state of polarization. This was consistent with the appearances that they exhibited, as SC8 exhibited symptoms of deterioration, whereas RYG1 did not, after 21 days of storage. Consistently with the results of the principal component analysis, only a small quantity of differentially expressed genes (68 DEGs) was detected in the comparison between FR and FS, indicating that both of them had similar gene expression profiles when fresh. However, after 21 days of storage, a great deal of differentially expressed genes were generated in SR vs. SS, SR vs. FR and SS vs. FS, indicating that RYG1 and SC8 had substantially different responses to PPD at the gene transcription level. The 2008 co-DEGs shared in SR vs. SS with either SR vs. FR or SS vs. FS were considered to be the most important genes mediating a series of defense mechanisms against PPD in RYG1 cassava.

In previous studies, it has been shown that reactive oxygen species (ROS) are active during cassava deterioration [30,36]. Indeed, they have been considered as the main cause of PPD. Thus, the genes responsible for scavenging reactive oxygen species and their regulatory mechanisms have become the focus of research. In this study, we also found that some upregulated co-DEGs were enriched in the GO terms annotated as the response to reactive oxygen species (Table S2, Figure S1). Most of the genes were described as *HSP20s*. Moreover, these genes were upregulated or unchanged in stored RYG1 compared to the fresh control; however, most of these genes were downregulated in stored SC8 compared to its fresh control (Table S4). In this study, we also found that ROS fluorescence and H_2_O_2_ content in 21-day-storage RYG1 cassava were significantly lower than those in 21-day-storage SC8 cassava (Figures S2 and S3). Therefore, we hypothesized that the positive expression of these reactive oxygen species responsive genes in stored RYG1 triggers a series of reactive oxygen species scavenging reactions, thereby maintaining intracellular reactive oxygen species stability without causing cell toxicity.

Cell wall integrity and its regulatory genes have often been considered in relation to the storage tolerance of agricultural products after harvest [37,38,39]. High-CO_2_ treatment increases the pectin content and hardness of strawberries, and reduces their rate of rotting. At the same time, the expression of genes related to cell wall degradation, such as pectin methylesterase (PME), polygalacturonase (PG) and pectin lyase (PL), is inhibited [37]. The rapid softening of peach flesh was found to occur due to the stronger expression of genes encoding cell wall hydrolases, especially xylosidases and galactosidases [38]. Furthermore, inhibition of the expression of cell wall modification genes related to fruit softening, such as *PG*, cellulase, *PME*, alpha-l-arabinofuranosidase and beta-galactosidase, can improve storage tolerance in grapefruits [39]. The differential expression of some genes related to cell wall remodeling, such as pectin methylesterase (*PME*) and α-mannosidases (*MNS*), has also been detected in cassava at the early stage of postharvest metamorphism [40]. In our study, transmission electron microscopy showed that the cell wall of SR was more intact than that of SS. GO functional annotation and enrichment analysis of co-DEGs revealed that many cell-wall-related genes were significantly enriched, including *PME/PMEIs*, *PLLs*, *CEL* and *EXPs* (Figure 6d). In addition, the genes in the cutin, suberin and wax biosynthesis were also enriched. Therefore, we hypothesized that maintaining cell wall integrity is another mechanism by which RYG1 resists PPD and maintains its shelf life. These cell-wall-associated co-DEGs therefore deserve attention and require functional verification.

There was a strong correlation between an increase in soluble sugar content and the extension of postharvest shelf life. For example, one of the mechanisms involved in improving the postharvest life of sweet cherry fruit via heating and calcium treatment is that the treatment increases the content of soluble sugar, especially galactose [41]. In potato, the soluble sugar accumulation after cold treatment served to maintain the quality of the crop during storage [28]. In a previous study of cassava, the soluble sugar content after storage in a PPD-resistant variety was higher than that in susceptible varieties [34]. In the current study, we found that some genes involved in the starch and sucrose metabolism and galactose metabolism pathways were significantly enriched in the co-DEGs (Figure 6c). Moreover, the total soluble sugar content was higher in the PPD-tolerant RYG1 than in the control after 21 days storage (Figure S4), which is consistent with the previous study. On the other hand, the soluble sugar concentration is considered as a positive quality criterion in fruit, and it determines the sweetness of the fruit [42,43]. Although reports on cassava’s soluble sugar content or sweetness in cassava quality are rare, there have been several reports that mention a preference for cassava with sweetness [44,45,46]. Since some (not all) prefer no taste in cassava products, the increase in soluble sugar content after storage may lead more people to favor cassava products, thus stimulating the cassava industry. Therefore, we speculate that the expression of related genes plays important roles in regulating the anti-PPD capabilities of RYG1. The hexokinase (HXK) activity and *HXK* gene expression in peach fruits were increased by postharvest disease stress [47]. Plant HXK can independently catalyze hexose phosphorylation into glycolysis and is sensitive to glucose. HXK phosphorylation of hexoses affects a variety of hormone signaling pathways, as well as plant development and stress responses [48]. In our study, one *HXK* was identified, and its expression was significantly decreased after storage in RYG1, while it was unaltered in SC8. This suggests that the sugar signal in RYG1 may have been sensed and thereby effectively regulated metabolic activity.

In addition to the above possible mechanisms of resistance to PPD, we also found that upregulated co-DEGs were mainly enriched in the photosynthetic antenna protein, photosynthesis and protein processing in the endoplasmic reticulum KEGG pathways (Table 1). Previous studies have shown that the activation of the expression of photosynthesis-related genes is closely related to the alleviation of the postharvest deterioration of agricultural products [49,50,51]. Light treatment delayed postharvest yellowing, retained chloroplast components and maintained slower sugar loss in broccoli [49]. Results in broccoli research indicate that postharvest treatments that delay chlorophyll degradation have a higher effect on the expression of pheophytinase genes [51]. Transcriptome analysis revealed that the mechanism by which low-intensity white LED light delayed postharvest pakchoi senescence was mainly through activating the expression of photosynthesis-related genes, including genes related to Photosystem II, Photosystem I, Cytochrome b6f complex, photosynthetic electron transport and F-type ATPase [50]. In this study, we found that the RYG1 cassava became light green after storage for 35 days (Figure 1). This result indicated that, after longtime storage, RYG1 began to produce and accumulate chlorophyll. This phenotypic change is also partially consistent with previous studies in other crops. Although the expression of photosynthesis-related genes was upregulated after storage in both RYG1 and SC8, the expression of these genes was higher after storage in RYG1 than in SC8 (Figure 6a). These results suggested that RYG1 may have a higher energy state due to a higher photosynthetic rate than that of SC8 occurring after storage, thus helping to delay the deterioration during postharvest storage. Interestingly, among the hub genes screened, we also found that eight hub genes (*PRK*, *LHCA3*, *PSAF*, *LHCA1*, *PETE*, *PSBY*, *PSAD-2* and *LHCA4*) were all related to photosynthesis (Figure 9bc). The direct involvement of these genes in delaying postharvest fruit deterioration has not been reported; however, several gene-related proteins have been reported as participating in the discoloration of postharvest fruits and vegetables [52,53]. The expression of *LHCI* and *LHCII* was significantly downregulated in the yellowing of broccoli heads [52]. The expression of LHC (CAB) proteins was downregulated with the decrease in the chlorophyll content in peach peel [53]. The *LHC* was also used as an index to measure tomato postharvest ripening [54]. This further piqued our interest, since it suggests that photosynthesis may play a non-negligible role in the PPD resistance of RYG1; however, given the fact that this observation was made in a tuberous root, rather than an above-ground organ of the plant, the exact physiological significance of this finding warrants further investigation. Nevertheless, the upregulation of photosynthesis in other below-ground tissues is not without precedence, with similar findings being reported for Acmella, Datura, Artmisia, Rudbeckia, Stevia, Tagetes, Alnus and cassava [55,56,57]. Long ago, researchers found that the chloroplast structure and chlorophyll fluorescence in green root plastids were similar to those in leaves, while there was no chloroplast structure formation in dark grown roots [55]. Moreover, these studies also showed that the green roots are photoautotrophic [55]. Although we have not detected the chloroplast structure in cassava tuberous roots, the green accumulation in cassava could be clearly found by observing 35-day cassava slices (Figure 1). In view of this, it is reasonable to speculate that cassava tuberous roots may form photoautotrophs under long-term light conditions after separation from the parent plant.

The genes involved in protein processing in the endoplasmic reticulum pathway were mainly heat shock protein (HSP) genes. HSPs are proteins encoded by multi-gene families that protect plants from abiotic stress and are produced under ultra-stable high temperatures. They act as molecular chaperones to maintain protein homeostasis in the cell by helping in protein folding, protein unfolding, protein assembly, protein intracellular distribution and protein degradation [58]. Environmental stresses often lead to protein dysfunction, so the ability of HSPs to repair and assist in the refolding of damaged proteins is important in order to protect cells from stress. It has been reported that heat shock proteins maintain an incredibly important role in reducing the postharvest sensitivity of fruits to biological and abiotic stresses [25,59]. For example, the molecular mechanism of extending the fruit shelf life and quality in citrus involves the upregulation of heat shock proteins [59]. Some upregulated *HSPs* were also detected in storage potato with good quality [28]. In our data, 23 *HSP20s*, 3 *HSP70s* and 2 *HSP90s* were identified in the co-DEGs. All of the *HSPs* were upregulated in SR compared SS. These *HSPs* were either upregulated or showed no significant difference in SR compared to FR. Meanwhile, in SS vs. FS, most of the *HSP* genes were downregulated (Figure 6b). As described in the above, *HSP20s* were also the major genes identified in this study in response to ROS. This highlights even further their importance in PPD resistance.

Phenolic secondary metabolites produced in the phenylpropanoid biosynthesis pathway affect the quality characteristics of plant foods, such as their appearance, flavor and other health properties [60]. Previous studies have identified that some proteins in the phenylpropanoid biosynthesis pathway respond to postharvest deterioration at the proteomic level [61]. In this work, genes involved in the phenylpropanoid biosynthesis and flavonoid biosynthesis pathways were specifically enriched in the downregulated co-DEGs in SR vs SS. It may be conjectured that the expression differences in these genes might be related to the maintenance of cassava root quality after harvest.

Phytohormone signaling pathways play important roles in many environmental stresses and are often involved in the storage and preservation of agricultural products [62,63]. The present transcriptome data implied the important role of auxin pathway genes in RYG1 resistant to PPD. Among the 38 genes involved in the plant hormone signal transduction pathway, 17 genes belonged to the auxin signal (Figure 7). Specifically, there were seven *AUX/IAAs* and three *ARFs* that were upregulated in SR vs SS. In strawberry, exogenous IAA delayed the fruit ripening process by upregulating the *AUX/IAA* and *ARF* genes and downregulating the genes related to cell wall degradation and pectin [22]. In the present data, most of the cell wall degradation-related genes were downregulated in RYG1 after storage. These results suggested that the auxin signaling pathway plays an important role in the PPD resistance of RYG1.

The complexity of the molecular mechanism of RYG1’s resistance to PPD was further demonstrated by constructing a protein–protein interaction network. Different gene-regulatory pathways intertwine to synergistically resist the occurrence of postharvest metamorphism (Figure 9). Among the selected 16 hub genes, with the exception of eight genes related to photosynthesis, there were another eight hub genes, including *GUN4, GGR, ALDH3F1, CYP38*, *NR1*, two *GDH2s* and *LASPO*. GUN4 is one of the key regulators in the chlorophyll biosynthesis pathway [64]. The reduction in chlorophyll loss in Chinese cabbage under 21 days of light storage was related to the upregulation of this gene’s expression [65]. *GGR* is another gene participating in chlorophyll biosynthesis [66]. Thus, *GUN4* and *GGR* may be involved in the fight against PPD by regulating the chlorophyll synthesis process. The stress-induced ROS cause the excessive accumulation of toxic aldehydes in cells. ALDHs can convert toxic aldehydes to nontoxic carboxylic acids, thus reducing the damage to cells [67]. LASPO is considered pivotal to maintain NAD homeostasis [68]. GDH2 activates the catabolic oxidation of glutamate and provides a carbon skeleton for the citrate cycle (TCA cycle) [69]. It is also a key enzyme in NH_4_^+^ assimilation and is involved in various stresses [70]. It is well documented that the downregulation of *GDH* expression and the reduced GDH activity are correlated with a delay in the initiation of senescence or the extended shelf life of fruits as well as vegetables [71,72,73]. A decrease in NR activity coincided with the alleviation of postharvest pericarp browning in longan fruit [74]. However, enhanced *NR* gene expression appears in the accelerated softening of tomato fruits [75]. The abovementioned evidence partially supports the notion that the function of these hub genes might be involved in regulating postharvest deterioration in cassava. The molecular mechanisms of these hub genes affecting the shelf life of postharvest cassava need to be further studied through a series of experiments. For example, the effect of these genes on PPD resistance in cassava can be observed by overexpressing or suppressing their expression in cassava.

## 4. Materials and Methods

### 4.1. Plant Treatment and Tissue Sampling

Cassava (*Manihot esculenta* Crantz) cultivars SC8 and RYG1 were selected for this study, according to previous results [35] in which SC8 and RYG1 were identified as being sensitive and tolerant to PPD, respectively. Cassavas were harvested after 1-year planting from the experiment field in the city of Wenchang, Hainan Province, China, in March 2015. They were kept at 28~30 °C and 65~70% relative humidity, under 16/8 h light/dark conditions. The cassavas, with the proximal and distal ends removed, were transected weekly (0, 7, 14, 21, 28, 35 and 42 d) with a thickness of 1 cm, to detect the PPD in the sections.

### 4.2. Transmission Electron Microscope Observations

Cassava samples of FR, SR, FS and SS were fixed in glutaraldehyde (2.5%) for more than 3 h. The samples were cleaned 3–4 times with phosphoric acid buffer (PAB, PH7.2, 0.1 mol/L), and then fixed overnight with osmium acid (1%) at 4 °C. After this, the samples were washed with PAB (0.1 mol/L, pH 7.2) 3 to 4 times, for 45 min each time. Next, the samples were dehydrated with 50%, 70%, 80%, 90% and 100% ethanol solutions for 12 to 15 min each time, followed by epoxy propane 2 times, for 10 min each time, with epoxy propane:EPON resin = 2:1, and epoxy propane:EPON resin = 1:2. They were permeated for 2 h, and then placed in pure resin overnight. This was replaced with acetone:ethanol = 1:1 and pure acetone for 25–35 min each time, and then with acetone:EPON resin (3:1) for 2 h. We then placed samples in acetone:EPON resin (1:1) and permeated them for 2 h; in acetone:EPON resin (1:3), they were permeated for 2 h, and then in pure resin overnight. We then replaced the sample overnight with pure resin and then buried the sample in an embedded plate with pure resin. The polymerization was carried out at 37 °C for 12 h, 45 °C for 12 h and 60 °C for 48 h. We then sliced, dyed, dried and observed the samples.

### 4.3. RNA-Seq Transcriptome Analysis

The samples from 0 and 21 d treatments were collected and frozen with liquid nitrogen for the total RNA extraction. The methods of total RNA extraction and RNA-seq library conduction were same as those previously described [76]. The RNA quality and concentrations were evaluated using an Agilent 2100 Bioanalyzer (Agilent Technologies, Santa Clara, CA, USA). Equal amounts of RNA from each sample were sequenced on an Illumina HiSeq 2500 sequencer (Illumina, San Diego, CA, USA) at the Gene Denovo company (Guangzhou China), to construct the strand-specific RNA-seq libraries. The data were submitted to the National Genomics Data Center (NGDC) with the assigned accession number CRA007720. The experiments on RYG1 and SC8 were performed with two biological repeats, and each repeat consisted of a mixture of five individual roots.

### 4.4. Screening of Differentially Expressed Genes (DEGs) and Enrichment Analysis

RNA-seq data analysis was carried out essentially according to previous methods [76]. Briefly, the ribosomal RNA reads of raw data were first removed to obtain clean reads. The clean data were aligned to the cassava genome [77]. For each cassava gene, its expression level was calculated using the fragments per kilobase of transcript per million mapped reads (FPKM) method [78]. The R language package was used for principal component analysis (PCA). The FDR and |log2FC| were used to screen differentially expressed genes (DEGs). The screening conditions were FDR < 0.05 and |log2FC| ≥ 2. The co-DEGs were screened using a Venn diagram. The heatmaps of the selected co-DEGs were drawn with the program in Omicshare.

### 4.5. Enrichment Analysis, PPI Interaction Network Construction of co-DEGs

The GO enrichment analysis, KEGG pathway enrichment analysis and the interaction networks of co-DEGs were generated by the STRING online platform (https://cn.string-db.org/, 23 October 2022), with confidence scores higher than 0.7. In the PPI network, the hub genes were screened by the Degree algorithm of the CytoHubba plugin in an open-source software program, Cytoscape v 3.9.0 (https://cytoscape.org/, 6 November 2022) [79,80]. The genes with an interaction degree more than 15 were selected as hub genes in this study.

### 4.6. Quantitative Real-Time PCR Analysis

The PrimeScript RT reagent kit with gDNA Eraser was utilized to reverse the purified RNA of each sample to first-strand cDNA. The *Tubulin* gene in cassava was used as an internal control. All the primers used in this work were designed with Primer Premier 5. The primers are listed in Supplementary Table S5. QRT-PCR was performed with a SYBR^®^Premix Ex TaqTM II Kit and a ABI 7900HT Fast Real-Time PCR System (Applied Biosystems, Foster City, CA, USA). The relative expression levels of target genes were calculated via 2^−∆∆Ct^ relative quantitative analysis [76]. The data were statistically analyzed using GraphPad Prism 8.3.0.538 to generate bar charts.

## 5. Conclusions

This study suggested that the longer shelf life of RYG1 may be related to the maintenance of lower reactive oxygen species, higher soluble sugar levels and more intact cell wall structures. In addition, the regulation of photosynthesis and endoplasmic reticulum protein processing, phenolic secondary metabolites and phytohormone signaling pathways also plays an important role in the resistance to PPD. Moreover, we screened out some of the hub genes that connected these important biological processes, including *PRK*, *LHCA3*, *PSAF*, *LHCA1*, *PETE*, *PSBY*, *PSAD-2*, *LHCA4*, *GUN4*, *GGR*, *ALDH3F1*, *CYP38*, *NR1*, 2 *GDH2s* and *LASPO*. These findings provide targets for the further improvement of cassava storage tolerance.

## Figures and Tables

**Table 1:** KEGG pathway enrichment of co-DEGs.

	Classify	Pathway ID	Description	Gene Number	False Discovery Rate
upregulated	Energy metabolism	map00195	Photosynthesis	10	0.00024
		map00196	Photosynthesis—antenna proteins	6	0.00036
	Folding, sorting and degradation	map04141	Protein processing in endoplasmic reticulum	29	2.08 × 10^−9^
	Global and overview maps	map01110	Biosynthesis of secondary metabolites	51	0.0252
	Lipid metabolism	map00062	Fatty acid elongation	5	0.0418
		map00073	Cutin, suberine and wax biosynthesis	7	0.0021
	Signal transduction	map04075	Plant hormone signal transduction	18	0.0156
downregulated	Amino acid metabolism	map00250	Alanine, aspartate and glutamate metabolism	9	0.0058
		map00270	Cysteine and methionine metabolism	23	9.50 × 10^−7^
		map00400	Phenylalanine, tyrosine and tryptophan biosynthesis	9	0.0061
	Biosynthesis of other secondary metabolites	map00940	Phenylpropanoid biosynthesis	42	8.91 × 10^−14^
		map00941	Flavonoid biosynthesis	8	0.034
		map00945	Stilbenoid, diarylheptanoid and gingerol biosynthesis	8	0.0082
	Carbohydrate metabolism	map00052	Galactose metabolism	9	0.0153
		map00500	Starch and sucrose metabolism	16	0.0136
		map00520	Amino sugar and nucleotide sugar metabolism	14	0.0315
	Global and overview maps	map01100	Metabolic pathways	211	2.41 × 10^−21^
		map01110	Biosynthesis of secondary metabolites	149	2.07 × 10^−22^
		map01200	Carbon metabolism	27	0.0041
		map01230	Biosynthesis of amino acids	27	0.00043
	Metabolism of other amino acids	map00450	Selenocompound metabolism	5	0.0282
		map00460	Cyanoamino acid metabolism	9	0.0035
		map00480	Glutathione metabolism	24	4.65 × 10^−9^
	Metabolism of terpenoids and polyketides	map00900	Terpenoid backbone biosynthesis	13	0.00043
		map00904	Diterpenoid biosynthesis	6	0.03
	Signal transduction	map04016	MAPK signaling pathway—plant	24	2.89 × 10^−5^

## Data Availability

Not applicable.

## Supplementary Materials

The following supporting information can be downloaded at: https://www.mdpi.com/article/10.3390/ijms24010246/s1.
