# Supplementary material for: Comparative Transcriptome Profiling of Cassava Tuberous Roots in Response to Postharvest Physiological Deterioration
Source: Int J Mol Sci. 2022 Dec 23;24(1):246. doi: 10.3390/ijms24010246 (PMC9820078; doi:10.3390/ijms24010246)
Supplement: Supplementary file 1 [file ijms-24-00246-s001.zip › Figures.pdf]

(a)

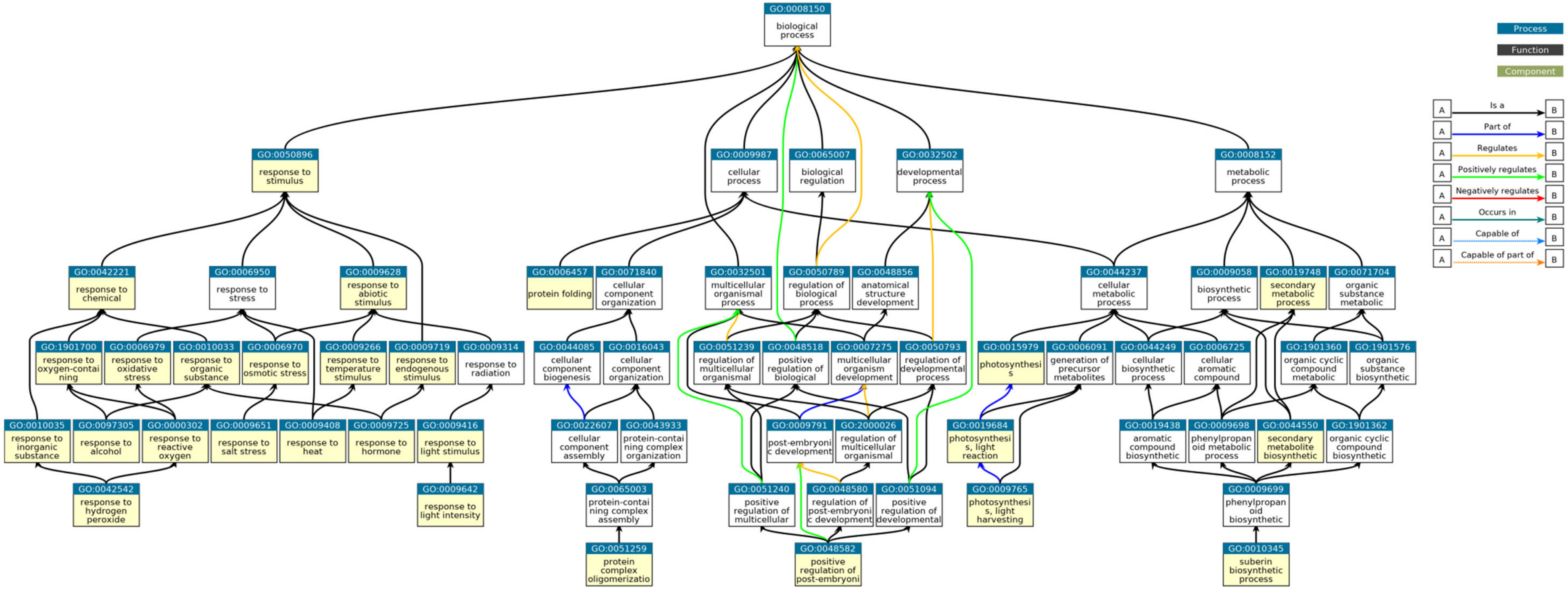
GO:0008150 biological process
GO:0050896 response to stimulus
GO:0009987 cellular process
GO:0065007 biological regulation
GO:0032502 developmental process
GO:0008152 metabolic process
GO:0042221 response to chemical
GO:0006950 response to stress
GO:0009628 response to abiotic stimulus
GO:0006457 protein folding
GO:0071840 cellular component organization
GO:0032501 multicellular organismal process
GO:0050789 regulation of biological process
GO:0048856 anatomical structure development
GO:0044237 cellular metabolic process
GO:0009058 biosynthetic process
GO:0019748 secondary metabolic process
GO:0071704 organic substance metabolic
GO:1901700 response to oxygen-containing
GO:0006979 response to oxidative stress
GO:0010033 response to organic substance
GO:0006970 response to osmotic stress
GO:0009266 response to temperature stimulus
GO:0009719 response to endogenous stimulus
GO:0009314 response to radiation
GO:0044085 cellular component biogenesis
GO:0016043 cellular component organization
GO:0051239 regulation of multicellular organismal
GO:0048518 positive regulation of biological
GO:0007275 multicellular organism development
GO:0050793 regulation of developmental process
GO:0015979 photosynthesis
GO:0006091 generation of precursor metabolites
GO:0044249 cellular biosynthetic process
GO:0006725 cellular aromatic compound
GO:1901360 organic cyclic compound metabolic
GO:1901576 organic substance biosynthetic
GO:0010035 response to inorganic substance
GO:0097305 response to alcohol
GO:0000302 response to reactive oxygen
GO:0009651 response to salt stress
GO:0009408 response to heat
GO:0009725 response to hormone
GO:0009416 response to light stimulus
GO:0022607 cellular component assembly
GO:0043933 protein-containing complex organization
GO:0009791 post-embryonic development
GO:2000026 regulation of multicellular organismal
GO:0019684 photosynthesis, light reaction
GO:0019438 aromatic compound biosynthetic
GO:0009698 phenylpropanoid metabolic process
GO:0044550 secondary metabolite biosynthetic
GO:1901362 organic cyclic compound biosynthetic
GO:0042542 response to hydrogen peroxide
GO:0009642 response to light intensity
GO:0065003 protein-containing complex assembly
GO:0051240 positive regulation of multicellular
GO:0048580 regulation of post-embryonic development
GO:0051094 positive regulation of developmental
GO:0009765 photosynthesis, light harvesting
GO:0009699 phenylpropanoid biosynthetic
GO:0051259 protein complex oligomerizatio
GO:0048582 positive regulation of post-embryoni
GO:0010345 suberin biosynthetic process
Process
Function
Component
A Is a B
A Part of B
A Regulates B
A Positively regulates B
A Negatively regulates B
A Occurs in B
A Capable of B
A Capable of part of B

(b)

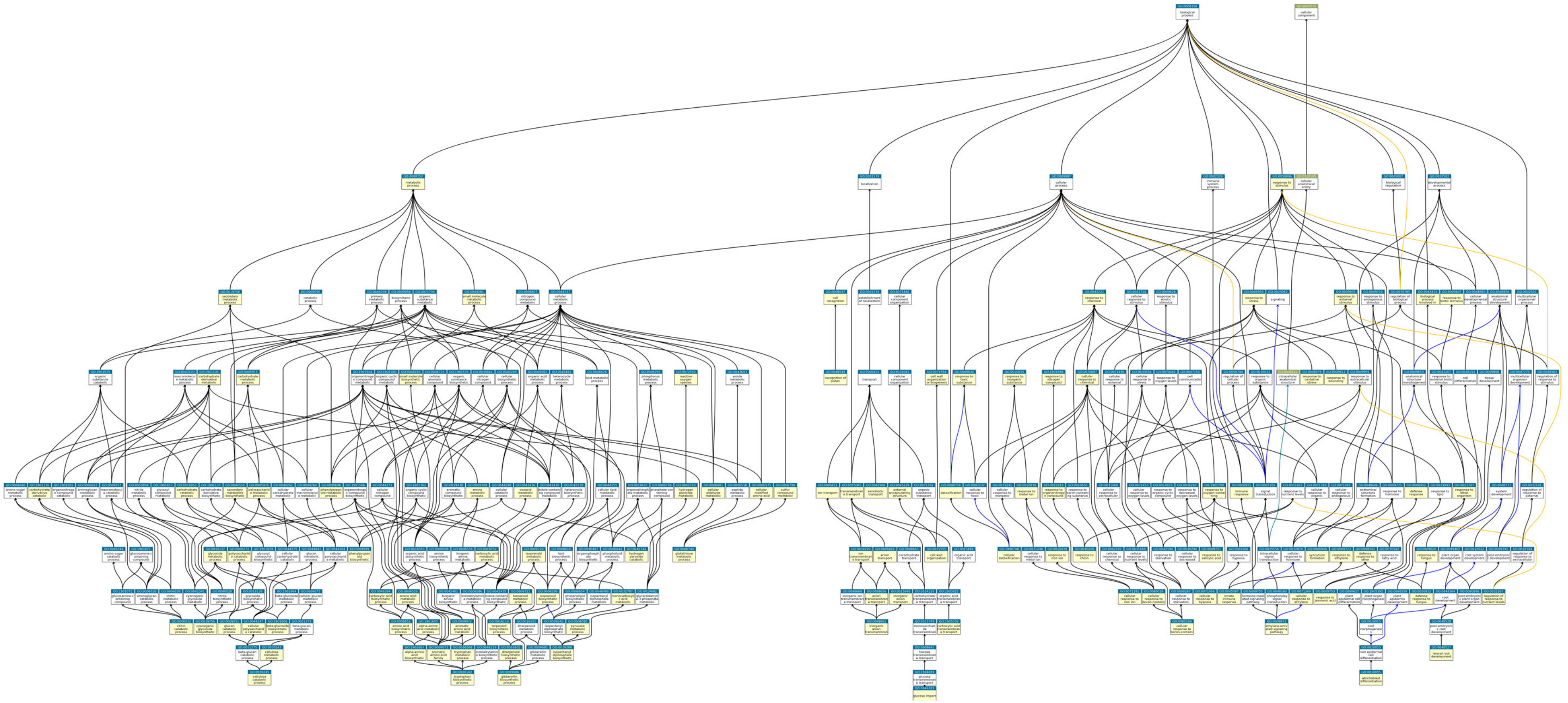

Figure S1. GO enrichment analysis of upregulated (a) and downregulated(b) co-DEGs in biological process terms

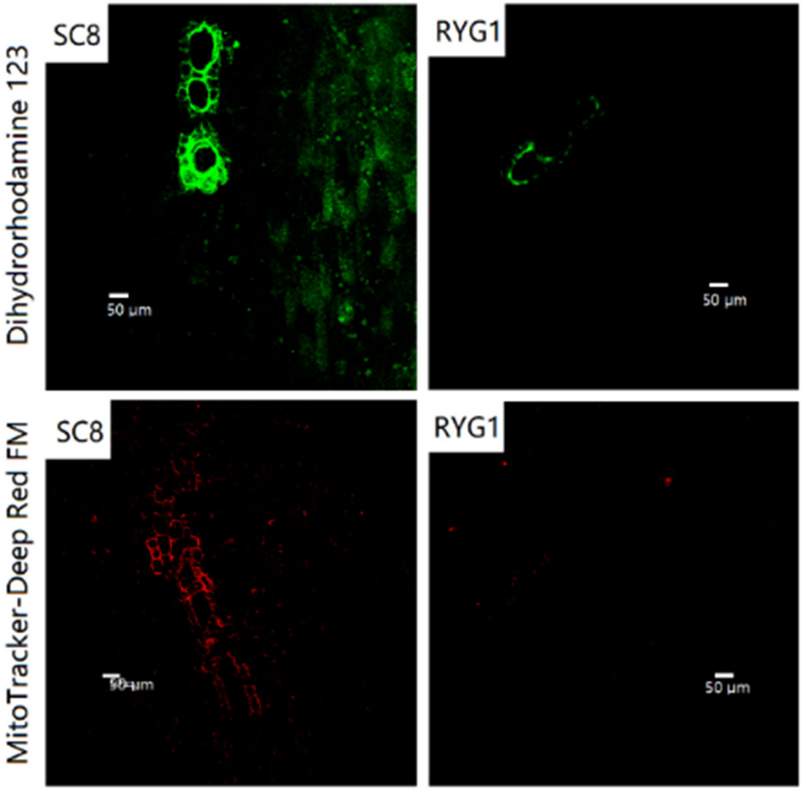

Figure S2. Fluorescence observation results showing reactive oxygen species production after 21-day- storage in RYG1 and SC8.

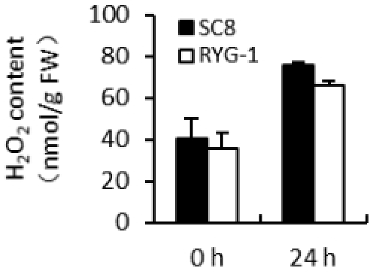

Figure S3. H_2O_2 content in 21-day-storage RYG1 and SC8 cassava.

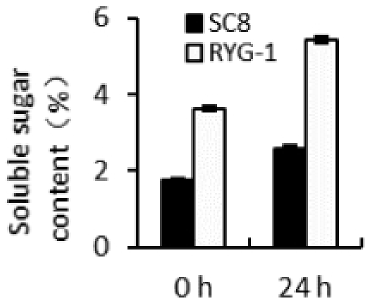

Figure S4. Soluble sugar content in 21-day-storage RYG1 and SC8 cassava.
