# Supplementary material for: Comparative Transcriptome Profiling of Cassava Tuberous Roots in Response to Postharvest Physiological Deterioration
Source: Int J Mol Sci. 2022 Dec 23;24(1):246. doi: 10.3390/ijms24010246 (PMC9820078; doi:10.3390/ijms24010246)
Supplement: Supplementary file 1 [file ijms-24-00246-s001.zip › Table S1.pdf]

Table S1 Classification of data of different samples.

Sample	Total Raw Reads (bp)	Total Clean Reads(bp)	Total Clean Reads(Gb)	Q20(%)	Q30(%)	Clean Reads Ratio (%)	Total Clean Reads Num	Total reads without rRNA	Total Mapping (%)	Uniquely Mapping (%)
F-SC8-1	3,194,578,500	3,163,875,500	3.16	95.72%	91.57%	99.04%	25,311,004	24,271,212	81.63%	81.42%
F-SC8-2	3,085,361,750	3,054,025,250	3.05	95.39%	90.88%	98.98%	24,432,202	19,621,306	77.75%	77.56%
S-SC8-1	3,179,155,750	3,146,779,250	3.15	95.44%	91.09%	98.98%	25,174,234	23,688,878	80.22%	79.94%
S-SC8-2	2,984,153,000	2,952,679,750	2.95	95.37%	90.94%	98.95%	23,621,438	23,135,938	73.92%	73.57%
F-RYG1-1	2,829,267,000	2,796,811,500	2.80	95.43%	91.06%	98.85%	22,374,492	21,927,792	77.74%	77.53%
F-RYG1-2	3,032,646,000	3,004,534,750	3.00	95.60%	91.25%	99.07%	24,036,278	18,478,534	76.50%	76.33%
S-RYG1-1	2,805,897,500	2,774,660,250	2.77	95.29%	90.79%	98.89%	22,197,282	20,480,816	82.02%	81.77%
S-RYG1-2	2,805,081,250	2,778,187,250	2.78	95.63%	91.44%	99.04%	22,225,498	21,560,704	84.07%	83.73%
