# Supplementary material for: Comparative Transcriptome Profiling of Cassava Tuberous Roots in Response to Postharvest Physiological Deterioration
Source: Int J Mol Sci. 2022 Dec 23;24(1):246. doi: 10.3390/ijms24010246 (PMC9820078; doi:10.3390/ijms24010246)
Supplement: Supplementary file 1 [file ijms-24-00246-s001.zip › Table S2.pdf]

Table S2 GO enrichment analysis of upregulated and downregulated co-DEGs

Biological process GO terms

#term ID	term description	observed gene count	background gene count	strength	false discovery rate
**upregulated co-DEGs**					
GO:0050896	Response to stimulus	222	6678	0.15	######
GO:0042221	Response to chemical	128	3176	0.23	######
GO:0009628	Response to abiotic stimulus	100	1915	0.34	######
GO:0010033	Response to organic substance	81	2197	0.19	0.0205
GO:1901700	Response to oxygen-containing compound	78	1600	0.31	######
GO:0009719	Response to endogenous stimulus	71	1696	0.25	0.0016
GO:0009725	Response to hormone	69	1638	0.25	0.0017
GO:0009266	Response to temperature stimulus	47	567	0.54	######
GO:0009416	Response to light stimulus	41	622	0.45	######
GO:0010035	Response to inorganic substance	40	809	0.32	0.0063
GO:0009408	Response to heat	36	285	0.73	######
GO:0006979	Response to oxidative stress	36	484	0.5	######
GO:0006970	Response to osmotic stress	34	500	0.46	######
GO:0009651	Response to salt stress	31	405	0.51	######
GO:0006457	Protein folding	30	309	0.61	######
GO:0015979	Photosynthesis	27	223	0.71	######
GO:0097305	Response to alcohol	27	510	0.35	0.0384
GO:0000302	Response to reactive oxygen species	25	209	0.7	######
GO:0042542	Response to hydrogen peroxide	22	113	0.92	######
GO:0019748	Secondary metabolic process	22	287	0.51	0.0014
GO:0051259	Protein complex oligomerization	21	106	0.92	######
GO:0044550	Secondary metabolite biosynthetic process	15	192	0.52	0.0272
GO:0019684	Photosynthesis, light reaction	12	108	0.67	0.0068
GO:0009642	Response to light intensity	11	97	0.68	0.012
GO:0010345	Suberin biosynthetic process	10	33	1.11	######
GO:0048582	Positive regulation of post-embryonic develc	9	72	0.72	0.0272
GO:0009765	Photosynthesis, light harvesting	8	37	0.96	0.0026
**downregulated**					
GO:0008152	Metabolic process	677	13896	0.07	######
GO:0050896	Response to stimulus	364	6678	0.11	######
GO:0006950	Response to stress	246	3733	0.2	######
GO:0055114	Oxidation-reduction process	225	2314	0.37	######
GO:0042221	Response to chemical	223	3176	0.22	######
GO:0044281	Small molecule metabolic process	153	2184	0.22	######
GO:0070887	Cellular response to chemical stimulus	138	1943	0.23	######
GO:0055085	Transmembrane transport	130	1668	0.27	######
GO:0006952	Defense response	123	1532	0.28	######
GO:0009605	Response to external stimulus	118	1478	0.28	######
GO:0043436	Oxoacid metabolic process	117	1432	0.29	######
GO:1901700	Response to oxygen-containing compound	110	1600	0.22	0.0001
GO:0019752	Carboxylic acid metabolic process	106	1318	0.28	######
GO:0009607	Response to biotic stimulus	102	1076	0.36	######
GO:0044419	Interspecies interaction between organisms	99	1146	0.31	######
GO:0006811	Ion transport	95	1288	0.25	######
GO:0051707	Response to other organism	94	1031	0.34	######
GO:0005975	Carbohydrate metabolic process	92	1418	0.19	0.0045
GO:0098542	Defense response to other organism	77	801	0.36	######
GO:0034220	Ion transmembrane transport	74	996	0.25	0.0006
GO:0044283	Small molecule biosynthetic process	72	973	0.25	0.0009
GO:1901135	Carbohydrate derivative metabolic process	64	997	0.19	0.0463
GO:0010035	Response to inorganic substance	62	809	0.26	0.0013
GO:0046394	Carboxylic acid biosynthetic process	61	716	0.31	0.0001
GO:0071554	Cell wall organization or biogenesis	58	819	0.23	0.0117
GO:0006820	Anion transport	53	520	0.39	######
GO:0032787	Monocarboxylic acid metabolic process	51	692	0.25	0.0117
GO:0045229	External encapsulating structure organizatio	50	704	0.23	0.0243
GO:0071555	Cell wall organization	48	679	0.23	0.0331
GO:0005976	Polysaccharide metabolic process	45	588	0.26	0.0124
GO:0098656	Anion transmembrane transport	44	383	0.44	######
GO:0006520	Cellular amino acid metabolic process	44	493	0.33	0.0009
GO:0006790	Sulfur compound metabolic process	43	379	0.43	######
GO:0006979	Response to oxidative stress	41	484	0.31	0.0043
GO:0009723	Response to ethylene	39	343	0.43	######
GO:0016052	Carbohydrate catabolic process	38	409	0.35	0.0015
GO:1901605	Alpha-amino acid metabolic process	37	368	0.38	0.0005
GO:0071369	Cellular response to ethylene stimulus	35	278	0.48	######
GO:0009636	Response to toxic substance	35	294	0.45	######
GO:0009620	Response to fungus	34	329	0.39	0.0007
GO:0009873	Ethylene-activated signaling pathway	33	271	0.46	######
GO:0019748	Secondary metabolic process	33	287	0.44	0.0001
GO:1901698	Response to nitrogen compound	33	386	0.31	0.014
GO:0050832	Defense response to fungus	31	259	0.46	0.0001
GO:0098754	Detoxification	30	231	0.49	######
GO:0008652	Cellular amino acid biosynthetic process	29	259	0.43	0.0008
GO:0006720	Isoprenoid metabolic process	29	292	0.38	0.0044
GO:0010243	Response to organonitrogen compound	29	337	0.31	0.0256
GO:0072593	Reactive oxygen species metabolic process	28	171	0.59	######
GO:1990748	Cellular detoxification	28	211	0.5	######
GO:0006721	Terpenoid metabolic process	28	245	0.44	0.0008
GO:0008299	Isoprenoid biosynthetic process	28	252	0.42	0.0012
GO:0000272	Polysaccharide catabolic process	28	260	0.41	0.0019
GO:0006955	Immune response	28	339	0.3	0.0495
GO:0016114	Terpenoid biosynthetic process	27	208	0.49	0.0002
GO:0010038	Response to metal ion	27	279	0.36	0.0095
GO:0045087	Innate immune response	26	305	0.31	0.0492
GO:0006575	Cellular modified amino acid metabolic proc	25	119	0.7	######
GO:0010200	Response to chitin	25	147	0.61	######
GO:0015698	Inorganic anion transport	24	164	0.54	0.0001
GO:1901607	Alpha-amino acid biosynthetic process	24	229	0.4	0.0078
GO:0042743	Hydrogen peroxide metabolic process	22	137	0.58	######
GO:0044550	Secondary metabolite biosynthetic process	22	192	0.44	0.0051
GO:0006749	Glutathione metabolic process	21	63	0.9	######
GO:0009751	Response to salicylic acid	20	123	0.59	0.0002
GO:0042744	Hydrogen peroxide catabolic process	20	129	0.57	0.0004
GO:0009611	Response to wounding	20	182	0.42	0.0138
GO:0009753	Response to jasmonic acid	20	187	0.41	0.0179
GO:0009072	Aromatic amino acid family metabolic proce	19	101	0.65	######
GO:0071456	Cellular response to hypoxia	18	142	0.48	0.0069
GO:1905039	Carboxylic acid transmembrane transport	18	173	0.4	0.0408
GO:0009698	Phenylpropanoid metabolic process	17	161	0.4	0.0465
GO:0042908	Xenobiotic transport	16	93	0.61	0.0009
GO:0009308	Amine metabolic process	16	146	0.42	0.0462
GO:1901136	Carbohydrate derivative catabolic process	15	107	0.53	0.009
GO:0006090	Pyruvate metabolic process	15	122	0.47	0.0247
GO:0009699	Phenylpropanoid biosynthetic process	15	129	0.44	0.0398
GO:0008037	Cell recognition	14	86	0.59	0.0044
GO:0016102	Diterpenoid biosynthetic process	14	100	0.52	0.0137
GO:0009073	Aromatic amino acid family biosynthetic proc	13	69	0.65	0.0024
GO:0030243	Cellulose metabolic process	13	82	0.58	0.0088
GO:0009686	Gibberellin biosynthetic process	13	93	0.52	0.0207
GO:0048544	Recognition of pollen	12	74	0.59	0.012
GO:0098661	Inorganic anion transmembrane transport	12	77	0.57	0.0151
GO:0048527	Lateral root development	12	82	0.54	0.0233
GO:0006081	Cellular aldehyde metabolic process	11	74	0.55	0.0334
GO:0010039	Response to iron ion	10	28	0.93	0.0003
GO:0006032	Chitin catabolic process	10	43	0.74	0.0043
GO:0046323	Glucose import	10	52	0.66	0.0122

Cellular components

#term ID	term description	observed gene count	background gene count	strength	false discovery rate
**upregulated**					
GO:00480	Apoplast	48	396	0.46	1.59E-07
GO:01101	Cellular anatomical entity	1067	23375	0.04	1.59E-07
GO:00055	Extracellular region	160	1792	0.33	3.35E-15
GO:00095	Photosystem	13	91	0.53	0.0216
GO:00095	Photosystem i	10	37	0.81	0.0017
GO:00719	Cell periphery	280	4866	0.14	3.93E-06
GO:00056	Cell wall	58	692	0.3	0.00026
GO:00160	Integral component of me	305	5316	0.14	1.12E-06
GO:00312	Intrinsic component of me	323	5544	0.15	1.52E-07
GO:00312	Intrinsic component of pla	37	424	0.32	0.0055
GO:00160	Membrane	440	8895	0.07	0.0027
GO:00058	Plasma membrane	226	4198	0.11	0.0055
GO:00057	Vacuole	72	1067	0.21	0.0086
**downregulated**					
GO:00480	Apoplast	37	396	0.43	8.61E-05
GO:01101	Cellular anatomical entity	885	23375	0.04	6.47E-06
GO:00055	Extracellular region	120	1792	0.29	2.41E-08
GO:00095	Photosystem	16	91	0.71	0.00012
GO:00095	Photosystem i	11	37	0.93	0.00011
GO:00095	Chloroplast	103	2010	0.17	0.007
GO:00095	Chloroplast thylakoid	33	414	0.36	0.0025
GO:00095	Chloroplast thylakoid men	28	355	0.36	0.0086
GO:00095	Photosystem i reaction ce	4	8	1.16	0.0353
GO:00095	Photosystem ii	12	71	0.69	0.0025
GO:00095	Plastid	117	2294	0.17	0.0029
GO:00095	Thylakoid	37	460	0.37	0.0011
GO:00426	Thylakoid membrane	32	376	0.39	0.0014

Molecular functions

#term ID	term description	observed gene count	background gene count	strength	false discovery rate
**upregulated**					
GO:00302	Carbohydrate binding	33	424	0.35	0.0062
GO:00038	Catalytic activity	524	####	0.12	4.71E-12
GO:00431	Cation binding	253	4912	0.17	3.47E-08
GO:00200	Heme binding	57	512	0.51	1.40E-10
GO:00055	Iron ion binding	37	444	0.38	0.00072
GO:00468	Metal ion binding	251	4876	0.17	3.75E-08
GO:00044	Monooxygenase activity	40	428	0.43	2.35E-05
GO:00164	Oxidoreductase activity	167	2078	0.37	1.80E-19
GO:00166	Oxidoreductase activity, acting on ch-c	24	303	0.36	0.0367
GO:00167	Oxidoreductase activity, acting on pair	48	524	0.42	2.43E-06
GO:00166	Oxidoreductase activity, acting on the	23	277	0.38	0.0288
GO:00046	Peroxidase activity	18	157	0.52	0.0054
GO:00436	Protein self-association	17	144	0.53	0.0059
GO:00469	Tetrapyrrole binding	65	552	0.53	4.87E-13
GO:00167	Transferase activity, transferring alkyl	18	176	0.47	0.0158
GO:00469	Transition metal ion binding	77	1420	0.2	0.0195
GO:00055	Calmodulin binding	24	305	0.36	0.0383
GO:00168	Carbon-oxygen lyase activity, acting o	9	55	0.67	0.0341
GO:00719	FAD binding	11	65	0.69	0.0074
GO:00053	Inorganic phosphate transmembrane t	7	29	0.84	0.0232
GO:00431	Ion binding	371	8664	0.09	0.00049
GO:00480	Monosaccharide binding	9	59	0.64	0.0465
GO:00166	Oxidoreductase activity, acting on perc	19	165	0.52	0.0034
GO:00198	Vitamin binding	17	184	0.43	0.0465
**downregulated**					
GO:00302	Carbohydrate binding	33	424	0.27	0.0445
GO:00038	Catalytic activity	608	####	0.1	2.69E-10
GO:00431	Cation binding	259	4912	0.1	0.0043
GO:00200	Heme binding	74	512	0.54	2.11E-15
GO:00055	Iron ion binding	53	444	0.46	4.22E-08
GO:00468	Metal ion binding	258	4876	0.1	0.0039
GO:00044	Monooxygenase activity	53	428	0.47	1.62E-08
GO:00164	Oxidoreductase activity	190	2078	0.34	2.45E-19
GO:00166	Oxidoreductase activity, acting on ch-c	32	303	0.4	0.00064
GO:00167	Oxidoreductase activity, acting on pair	60	524	0.44	1.62E-08
GO:00166	Oxidoreductase activity, acting on the	29	277	0.4	0.0017
GO:00046	Peroxidase activity	24	157	0.56	5.18E-05
GO:00436	Protein self-association	21	144	0.54	0.00035
GO:00469	Tetrapyrrole binding	81	552	0.55	3.30E-17
GO:00167	Transferase activity, transferring alkyl	25	176	0.53	9.06E-05
GO:00469	Transition metal ion binding	96	1420	0.21	0.0007
GO:00228	Active ion transmembrane transporter	32	352	0.34	0.0068
GO:00228	Active transmembrane transporter acti	73	752	0.37	8.13E-08
GO:00085	Anion transmembrane transporter acti	32	377	0.31	0.0181
GO:00162	Antioxidant activity	26	200	0.49	0.00018
GO:00152	Antiporter activity	24	251	0.36	0.0205
GO:00426	ATPase-coupled transmembrane trans	22	224	0.37	0.0237
GO:00151	Carbohydrate transmembrane transpo	14	97	0.54	0.011
GO:00053	Carbohydrate:proton symporter activity	10	55	0.64	0.018
GO:00080	Chitin binding	7	29	0.76	0.0277
GO:00045	Chitinase activity	9	43	0.7	0.015
GO:00512	Dioxygenase activity	25	262	0.36	0.018
GO:00037	DNA-binding transcription factor activit	109	1705	0.19	0.0015
GO:00465	Glucosyltransferase activity	31	262	0.45	0.00012
GO:00043	Glutathione transferase activity	18	67	0.81	1.50E-06
GO:00167	Hydrolase activity, acting on glycosyl b	45	630	0.23	0.0347
GO:00045	Hydrolase activity, hydrolyzing o-glyco	40	524	0.26	0.0235
GO:00151	Inorganic anion transmembrane transp	15	124	0.46	0.0274
GO:00153	Inorganic molecular entity transmembr	63	947	0.2	0.0234
GO:00301	Manganese ion binding	11	76	0.54	0.0358
GO:00167	Oxidoreductase activity, acting on pair	23	204	0.43	0.004
GO:00800	Quercetin 3-o-glucosyltransferase acti	10	62	0.59	0.0312
GO:00800	Quercetin 7-o-glucosyltransferase acti	10	61	0.59	0.0284
GO:00152	Secondary active transmembrane tran	50	487	0.39	7.48E-06
GO:00152	Solute:cation symporter activity	16	143	0.43	0.0352
GO:00152	Symporter activity	27	207	0.5	0.00012
GO:01401	Transcription regulator activity	111	1882	0.15	0.0181
GO:00167	Transferase activity, transferring glyco	65	756	0.31	3.25E-05
GO:00167	Transferase activity, transferring hexos	46	474	0.37	9.06E-05
GO:00228	Transmembrane transporter activity	123	1619	0.26	1.87E-07
GO:00052	Transporter activity	125	1726	0.24	1.59E-06
GO:00352	UDP-glucosyltransferase activity	25	213	0.45	0.0012
GO:00081	UDP-glycosyltransferase activity	41	403	0.39	0.0001
GO:00429	Xenobiotic transmembrane transporter	15	98	0.56	0.004

GO:0009251	Glucan catabolic process	10	58	0.62	0.0226
GO:0044247	Cellular polysaccharide catabolic process	9	49	0.64	0.0276
GO:0030245	Cellulose catabolic process	8	27	0.85	0.0053
GO:0006568	Tryptophan metabolic process	8	30	0.8	0.0088
GO:0080029	Cellular response to boron-containing substa	7	12	1.14	0.0008
GO:0019288	Isopentenyl diphosphate biosynthetic proces	7	14	1.08	0.0014
GO:0071281	Cellular response to iron ion	7	18	0.97	0.0043
GO:0016137	Glycoside metabolic process	7	33	0.71	0.0495
GO:0006949	Syncytium formation	6	13	1.04	0.0062
GO:0000162	Tryptophan biosynthetic process	6	18	0.9	0.0192
GO:1901806	Beta-glucoside biosynthetic process	6	22	0.81	0.0408
GO:0019756	Cyanogenic glycoside biosynthetic process	6	23	0.79	0.047
GO:0010055	Atrichoblast differentiation	5	5	1.38	0.0024
GO:0080169	Cellular response to boron-containing substa	5	5	1.38	0.0024
GO:0032107	Regulation of response to nutrient levels	5	10	1.08	0.0151
