# Supplementary material for: Comparative Transcriptome Profiling of Cassava Tuberous Roots in Response to Postharvest Physiological Deterioration
Source: Int J Mol Sci. 2022 Dec 23;24(1):246. doi: 10.3390/ijms24010246 (PMC9820078; doi:10.3390/ijms24010246)
Supplement: Supplementary file 1 [file ijms-24-00246-s001.zip › Table S3.pdf]

Table S3 Differencially expressed genes in plant hormone signal transduction

ID	SS vs FS	SR vs SS	SR vs FR	Description
02408m	-4.32	3.94		ARFs
02762m	-3.29	2.42		ARFs
02885m	-3.22	2.06		ARFs
02953m	-2.54	2.76		ARR-B
12135m	-2.04	4.17	2.09	AUX/IAAs
12530m	-4.36	4.42		AUX/IAAs
12706m		3.18	2.88	AUX/IAAs
14431m		5.21	14.49	AUX/IAAs
14900m	-6.10	2.37	-3.31	AUX/IAAs
15577m	10.50	3.61	14.11	AUX/IAAs
21897m		10.57	10.57	AUX/IAAs
06788m	4.29	-3.94		AUX1
12164m	3.92	-2.38		BKI1
06280m		-2.12	-2.56	BSK
09919m	-6.77	3.51	-3.30	CYCD3
28677m	-6.00	4.94		DELLA
				EIN2
15566m	3.96	-5.43		ERFs
15673m		-2.65	-2.71	ERFs
15691m	10.91	-10.91		ERFs
15856m	3.02	-5.70	-2.40	ERFs
22781m	3.68	-5.55	-2.85	ERFs
02375m	2.80	-2.33		ETR
03838m	-3.13	2.74		GH3s
04078m	-2.96	-4.30	-7.72	GH3s
04196m	-8.64	8.78		GH3s
04899m		-2.41	-4.48	JAR1
16821m		-4.67	-6.27	JAZs
17020m	-2.53	-3.58	-6.06	JAZs
07913m		2.33	3.83	PP2C
13309m		2.53	3.00	PP2C
31181m	2.55	-10.39		PP2C
15315m		-6.31	-4.68	PYLs
16054m		-5.04	-7.36	PYLs
17683m	2.07	-2.27		SAURs
20041m		-4.23	-2.75	SAURs
21479m		14.75	4.58	SAURs
22896m	5.27	-5.25		TGA
13286m	-2.29	-2.52	-5.10	XET
