# Supplementary material for: Comparative Transcriptome Profiling of Cassava Tuberous Roots in Response to Postharvest Physiological Deterioration
Source: Int J Mol Sci. 2022 Dec 23;24(1):246. doi: 10.3390/ijms24010246 (PMC9820078; doi:10.3390/ijms24010246)
Supplement: Supplementary file 1 [file ijms-24-00246-s001.zip › Table S4.pdf]

Table S4 co-DEGs in the GO term of response to reactive oxygen species

id	SS vs FS	SR vs SS	SR vs FR	Description
cassava4 18158m	-3.21	2.46		17.6 kDa class II heat shock protein
cassava4 02195m	2.57	2.23	3.81	ABC2 homolog 13
cassava4 12494m		-8.18	-5.57	ACC oxidase 1
cassava4 24509m		11.60	4.19	ascorbate peroxidase 2
cassava4 03515m	5.71	-3.26		ATP-binding cassette 14
cassava4 04899m		-2.41	-4.48	Auxin-responsive GH3 family protein
cassava4 10580m		-2.84	-3.59	BTB and TAZ domain protein 1
cassava4 06297m		9.47	9.47	catalase 2
cassava4 15578m	2.38	-2.87		copper/zinc superoxide dismutase 2
cassava4 16548m	-2.86	3.72		HSP20-like chaperones superfamily pro
cassava4 17871m	-3.42	4.14		HSP20-like chaperones superfamily pro
cassava4 17974m		2.93	2.61	HSP20-like chaperones superfamily pro
cassava4 18038m	-4.00	4.13		HSP20-like chaperones superfamily pro
cassava4 18093m	2.36	3.30	6.82	HSP20-like chaperones superfamily pro
cassava4 18131m	-2.22	4.38	2.03	HSP20-like chaperones superfamily pro
cassava4 18200m		3.51	3.87	HSP20-like chaperones superfamily pro
cassava4 19659m	-2.01	2.26		HSP20-like chaperones superfamily pro
cassava4 21306m		11.63	11.63	HSP20-like chaperones superfamily pro
cassava4 22774m	-11.65	11.63		HSP20-like chaperones superfamily pro
cassava4 22909m	-2.16	2.39		HSP20-like chaperones superfamily pro
cassava4 24326m	-4.50	8.95	4.14	HSP20-like chaperones superfamily pro
cassava4 25159m		4.98	6.23	HSP20-like chaperones superfamily pro
cassava4 26342m	-2.79	7.18	3.58	HSP20-like chaperones superfamily pro
cassava4 28591m	-3.88	4.30		HSP20-like chaperones superfamily pro
cassava4 28846m	-2.26	3.95		HSP20-like chaperones superfamily pro
cassava4 32436m		3.70	3.55	HSP20-like chaperones superfamily pro
cassava4 33525m	-2.93	5.95	2.52	HSP20-like chaperones superfamily pro
cassava4 01966m		-3.87	-5.77	mechanosensitive channel of small con
cassava4 03178m		2.07	2.09	Protein of unknown function (DUF3506)
cassava4 21761m		-3.91	-2.21	senescence associated gene 20
cassava4 17157m		2.70	2.03	temperature-induced lipocalin
cassava4 22886m	-4.62	3.87		UDP-Glycosyltransferase superfamily p

›tein
›tein
›tein
›tein
›tein
›tein
›tein
›tein
›tein
›tein
›tein
›tein
›tein
›tein
›tein
›tein
›tein
›tein
ductance-like 10
)

rotein
