# Supplementary material for: Comparative Transcriptome Profiling of Cassava Tuberous Roots in Response to Postharvest Physiological Deterioration
Source: Int J Mol Sci. 2022 Dec 23;24(1):246. doi: 10.3390/ijms24010246 (PMC9820078; doi:10.3390/ijms24010246)
Supplement: Supplementary file 1 [file ijms-24-00246-s001.zip › Table S5.pdf]

Table S5. The list of primers used in the study

Gene ID	Primer F	Primer R
cassava4.1_003934m.g	ACATCCTTTGAAAAGTGAACCG	TTTCAGCTTCTGGCAGTTAAAC
cassava4.1_005092m.g	ACTGCTTATGAAAGGTCAAGGA	ACCAGCGTAGTATCGAAATAGG
cassava4.1_005418m.g	GTTGTTAATCGACGAAGATGCA	GCAGAAATAACCTCAACGGATC
cassava4.1_006013m.g	GAGACAACTGAAGAAGCTTTGG	CGAGAAGCAAGAAACTGAAACA
cassava4.1_007957m.g	CATTGAATGTGGCTAAACCGAA	CAAACTTCTTGGCTTCTTCGAA
cassava4.1_010967m.g	CGTTTGCGCGCTTATATAGTAA	TCCAGAGTTGTGAAGATGTTGA
cassava4.1_012097m.g	ATCAAAAGCTATGGCGGATTTC	AGATCAAAGTGTTCGCTACAGA
cassava4.1_014454m.g	ATGAGTTTGTTGGGGATAAGGT	GCAACAACTTCCATGGTAGATC
Tubulin	GTGGAGGAACTGGTTCTGGA	TGCACTCATCTGCATTCTCC
